# Glutathione S-Transferase P1 (GSTP1) gene polymorphism increases age-related susceptibility to hepatocellular carcinoma

**DOI:** 10.1186/1471-2350-11-46

**Published:** 2010-03-24

**Authors:** Yao-Li Chen, Hsin-Shun Tseng, Wu-Hsien Kuo, Shun-Fa Yang, Dar-Ren Chen, Hsiu-Ting Tsai

**Affiliations:** 1 Department of Surgery, Changhua Christian Hospital, Changhua, Taiwan; 2 School of Medicine, Chung Shan Medical University, Taichung, Taiwan; 3 Department of Medicine, Armed-Force Taichung General Hospital, Taichung, Taiwan; 4 General Education Center, Central Taiwan University of Science and Technology, Taichung, Taiwan; 5 Institute of Medicine, Chung Shan Medical University, Taichung, Taiwan; 6 School of Nursing, Chung Shan Medical University, Taichung, Taiwan; 7 Department of Nursing, Chung Shan Medical University Hospital, Taichung, Taiwan

## Abstract

**Background:**

Hepatocellular carcinoma (HCC) is one of the most frequent malignant neoplasms in the world. Genetic polymorphism has been reported to be a factor increasing the risk of HCC. Phase II enzymes such as glutathione s-transferases (GSTP1, GSTA1) play important roles in protecting cells against damage induced by carcinogens. The aim of this study was to estimate the relationship of the GSTP1 and GSTA1 gene polymorphisms to HCC risk and clinico-pathological status.

**Methods:**

Polymerase chain reaction-restriction fragment length polymorphism (PCR-RFLP) was used to measure GSTP1 (A→G) and GSTA1 (C→T) gene polymorphisms in 386 healthy controls and 177 patients with HCC.

**Results:**

Neither gene polymorphism was associated with the clinico-pathological status of HCC and serum expression of liver-related clinico-pathological markers. No association between the GSTA1 gene polymorphism and HCC susceptibility was found. However, in the younger group, aged ≤ 57 years, individuals with AG or GG alleles of GSTP1 had a 2.18-fold (95%CI = 1.09-4.36; p = 0.02) and 5.64-fold (95%CI = 1.02-31.18; p = 0.04) risk, respectively, of developing HCC compared to individuals with AA alleles, after adjusting for other confounders.

**Conclusion:**

AG and GG alleles of GSTP1 gene polymorphisms may be considered as factors increasing the susceptibility to and risk of HCC in Taiwanese aged ≤ 57 years.

## Background

Hepatocellular carcinoma (HCC) is one of the common causes of cancer-related death worldwide [1,2], and is the second leading cause of cancer death in Taiwan [3]. Phase II enzymes such as glutathione s-transferases (GSTP1, GSTA1) have been suggested to play an important role in protecting cells against damage induced by carcinogens, through regulation of the conjugation of a wide range of xenobiotics for excretion of hydrophilic metabolites [4-6]. The isoenzymes of glutathione transferases, GSTP1 [7-10] and GSTA1 [7,8,11,12], were found in several mammalian species [7,8] and nontumorous liver tissues [9-12]. Significantly increased expression of GSTP was demonstrated in early hepatocarcinogenesis [12] and HCC specimens [13], compared to their adjacent normal tissues or liver cirrhosis tissues. Loss of GSTP1 has been suggested to increase the risk of DNA damage and mutation [14,15]. An aberrant hypermethylation of GSTP1 promoter [9,10,16] and a subsequently induced lack of GSTP1 mRNA or protein expression were demonstrated in liver cancer cell lines [16], and in more than 77.8% of HBV-associated HCC tissues [9], respectively. Moreover, up-expression of GSTA was suggested to protect liver cells against oxidative stress [17,18] via an extracellular signal-regulated kinases (ERKs) and p38 kinase (p38K)-related pathway [18], as well as through the inhibition of H_2_O_2_-induced apoptosis to inhibit reactive oxygen species (ROS)-induced lipid peroxidation [17].

It was suggested that inactivated or down-regulated GSTP1 [4,5,9,14,15,19-22] and GSTA1 [23] genes could increase genomic damage when individuals were exposed to carcinogens. The GSTP1 gene, encoding glutathione S-transferase-pi and located on chromosome 11q13, was found with a single nucleotide substitution (A→G) at position 313, which resulted in replacing isoleucine (Ile) with valine (Val), substantially reduced GSTP1 enzyme activity [24,25], and induced slightly higher adduct levels in liver tissues [26]. Moreover, a wild genotype C at -69 in the promoter region of the human GSTA1 gene, designated as GSTA1* A, was substituted for T, designated as GSTA1*B, which resulted in decreasing GSTA1 enzyme expression or activity [11,27,28]. We therefore hypothesized that the phase II glutathione S-transferase (GST) genes, GSTP1 and GSTA1, were sensitive marker enzymes for preneoplastic and neoplastic liver cells, and genetic polymorphisms of these two enzymes could facilitate the susceptibility to and clinico-pathological development of HCC among Taiwanese, because more than 67% of male HCC patient and 55.2% of female HCC patient in Taiwan is related to hepatitis B virus (HBV) infection and HCV hepatitis C virus (HCV) infection, respectively [29], which are considered to be the important risk factors of HCC [26,30,31]; genetic polymorphisms of GSTP1 and GSTA1 could decrease the function of detoxification [11,24,25,27,28] when individuals are exposed to those risk factors. However, the roles of GSTP1 and GSTA1 gene polymorphisms in the susceptibility to HCC among Taiwanese have not been fully clarified. The purpose of this study was to investigate the influence of single nucleotide polymorphisms (SNPs) of the phase II GSTs, GSTP1 and GSTA1, on the susceptibility to and clinico-pathological development of HCC among Taiwanese.

## Methods

### Subjects and specimen collection

This was a hospital-based case-control study. A total of 177 patients with HCC diagnosed at Chung Shan University Hospital, Taichung, or Changhua Christian Hospital, Changhua, Taiwan, were recruited as a case group between April 2006 and August 2009. The diagnoses of HCC were according to the characteristic criteria of the national guidelines for HCC [32], and included liver injury diagnosed by either histology or cytology irrespective of a-fetoprotein (AFP) titer, in which imaging data showed any one of the following three cases: 1) one or more liver masses more than or equal to 2 cm in diameter via both computed tomography (CT) and magnetic resonance imaging (MRI); 2) one imaging data with early enhancement and a level of AFP more than or equal to 400 ng/mL; and 3) one imaging data with early arterial phase contrast enhancement plus early venous phase contrast washout regardless of AFP level. A total of 386 healthy controls were selected from those who visited the Department of Family Medicine, Chung Shan Medical University Hospital, Taiwan for health examination, based on not having a risk related to hepatocellular carcinoma and matched on demographic data of race, ethnic group, gender, and residential area. The whole blood specimens collected from the healthy controls and HCC patients were placed in tubes containing EDTA and immediately centrifuged and stored at -80°C. Associated clinico-pathological characteristics, such as HBsAg, anti-HCV, liver cirrhosis history, Child-Pugh grade, AFP, aspartate aminotransferase (AST), alanine aminotransferase (ALT), and stage of HCC, were verified by chart review. This study was performed with the approval of the Chung Shan University Hospital Institutional Review Board, and written informed consent was obtained from each individual.

### Genomic DNA extraction

Venous blood obtained from each subject was drawn into Vacutainer tubes containing EDTA and stored at 4°C. Genomic DNA was extracted using a commercial kit for extracting blood DNA (Qiagen, Valencia, USA), according to the manufacture's instructions. DNA was dissolved in TE buffer [10 mM Tris (PH 7.8), 1 mM EDTA] and then quantitated by a measurement of OD260. The final preparation was stored at -20°C and used as a template for polymerase chain reaction (PCR).

### Polymerase chain reaction-restriction fragment length polymorphism (PCR-RFLP)

The GSTP1 and GSTA1 gene variations test and the primers were modified [33]. The gene detections were amplified by PCR. Briefly, PCR was performed in a 10 μl reaction containing 100 ng DNA template, 1.0 μl of 10× PCR buffer (Invitrogen, Carslbad, CA, USA), 0.25 U of Taq DNA polymerase (Invitrogen, Carslbad, CA, USA), 0.2 mM dNTPs (Promega, Madison, WI, USA), and 200 nM of each primer (MDBio Inc. Taipei, Taiwan). The PCR cycling started at 94°C for 5 min followed by 35 cycles of 94°C for 1 min, 60°C for 1 min and 72°C for 2 min, with a final step at 72°C for 20 min to allow a complete extension of all PCR fragments. PCR products of the GSTP1 gene polymorphism were subjected to enzymatic digestion by incubation with BsmA1 for 4 hr at 37°C and then electrophoresis in 2% agarose gels. Wild type homozygous alleles (I/I) yielded 329- and 104-bp products, the heterozygous alleles (I/V) yielded 329-, 222-, 107- and 104-bp products, while the mutated type homozygous alleles (V/V) yielded a 222-, 107- and 104-bp products. PCR products of the GSTA1 gene polymorphism were subjected to enzymatic digestion by incubation with HinfI for 4 hr at 37°C and then electrophoresis in 2% agarose gels. Wild type homozygous alleles (A/A) yielded 525- and 254-bp products, the heterozygous alleles (A/B) yielded 525-, 254- and 197-bp products, while the mutated type homozygous alleles (B/B) yielded 525- and 197-bp products.

### Power and sample size calculations

Based on the hypothesis and our preliminary study, the frequencies of at least one mutated allele of GSTP1 and GSTA1 were 29.5% (114/386) and 21.2% (82/386), respectively. Assuming a 95% confidence interval, a p value of 0.05, a ratio of cases to healthy controls of 1:2, and at least 90% power to detect a 2.0-fold risk in gene variances of GSTP1 and GSTA1, the sample sizes were 150 and 175 case samples for gene polymorphisms of GSTP1 and GSTA1, respectively.

### Statistical analysis

The distributions of demographic characteristics and genotype frequencies between the cases and controls and the clinico-pathological features in different genotypes were analyzed by Fisher's exact test, since the small sample size was present in some categories of variables. The odds ratios (ORs) with their 95% confidence intervals (CIs) of the association between genotype frequencies and HCC were estimated by multiple logistic regression models, after controlling for other covariates, including age, gender and genotypes for each estimated variable. A non-parametric method was used because of the non-normal distribution of some estimated variables. The Mann-Whitney U test was used between two groups, and a Kruskal-Wallis test was used among three groups. Hardy-Weinberg equilibrium was assessed using a goodness-of-fit χ^2 ^test for bi-allelic markers. A *p *value of less than 0.05 was considered significant. The data were analyzed on SAS statistical software (Version 9.1, 2005; SAS Institute Inc., Cary, NC).

## Results

The frequency distributions of GSTP1 and GSTA1 gene polymorphisms were studied in 177 HCC patients and compared to 386 healthy controls. Both gene frequencies were in Hardy-Weinberg equilibrium (GSTP1: p > 0.05, χ^2 ^value: 0.48; GSTA1: p > 0.05, χ^2 ^value: 0.17, respectively) in our recruited healthy control group.

The demographic characteristics and the two gene polymorphism distributions in the HCC patients and healthy controls are shown in Table 1. Except for age *(p < 0.0001)*, there was a non-significant difference in distribution of gender, race, ethnic group, residential area, and genetic polymorphisms between the HCC patients and healthy controls, even when we further classified the individuals with at least one mutated allele as one subgroup and regarded the individuals with homozygous wild type alleles as another subgroup to estimate the adjusted odds ratios (AORs) with their 95% CIs, or combined different genetic distributions for estimating the interaction effect between the GSTP1 and GSTA1 gene polymorphisms on the susceptibility to HCC (Table 1).

**Table 1:** Adjusted odds ratio (AOR) and 95% confidence intervals (CIs) of hepatocellular carcinoma associated with genotypic frequencies and demographic characteristics distributions.

Variable	Controls (n = 386) (%)	Patients (n = 177) (%)	OR (95% CI) p value	AOR (95% CI) p value
**GSTP1**				

II	272 (70.5%)	112 (63.3%)	1.00	1.00

IV	107 (27.7%)	58 (32.7%)	1.31 (0.8-1.9) p = 0.16	1.16 (0.7-1.7) p = 0.46

VV	7 (1.8%)	7 (4.0%)	2.42 (0.8-7.0) p = 0.10	2.26 (0.7-7.1) p = 0.16

				

II	272 (70.5%)	112 (63.3%)	1.00	1.00

IV or VV	114 (29.5%)	65 (36.7%)	1.38 (0.9-2.0) p = 0.09	1.24 (0.8-1.8) p = 0.28

				

**GSTA1**				

AA	304 (78.8%)	146 (82.5%)	1.00	1.00

AB	78 (20.2%)	30 (16.9%)	0.80 (0.5-1.2) p = 0.34	0.89 (0.5-1.4) p = 0.66

BB	4 (1.0%)	1 (0.6%)	0.52 (0.05-4.7) p = 0.56	0.34 (0.04-3.5) p = 0.38

				

AA	304 (78.8%)	146 (82.5%)	1.00	1.00

AB or BB	82 (21.2%)	31 (17.5%)	0.78 (0.4-1.2) p = 0.36	0.85 (0.5-1.3) p = 0.52

				

**GST genes**				

Combination 1	212 (54.9%)	92 (52.0%)	1.00	1.00

Combination 2	152 (39.4%)	75 (42.3%)	1.13 (0.7-1.6) p = 0.51	1.05 (0.7-1.5) p = 0.77

Combination 3	22 (5.7%)	10 (5.7%)	1.04 (0.4-2.4) p = 0.81	1.08 (0.4-2.5) p = 0.84

				

**Age (yrs)**				

	54.37 ± 0.77	63.88 ± 0.81	p < 0.0001	

				

**Gender**				

Male	257 (66.6%)	128 (72.3%)	1.00	1.00

Female	129 (33.4%)	49 (27.7%)	0.76 (0.5-1.1) p = 0.17	1.08 (0.7-1.6) p = 0.72

				

Residential area	Mid-Taiwan	Mid-Taiwan		

Race	Asian	Asian		

Ethnicity	Taiwanese	Taiwanese		

The odds ratios (ORs) with their 95% CIs were estimated by logistic regression models.

The adjusted odds ratios (AORs) with their 95% CIs were estimated by multiple logistic regression models, after controlling for age, gender and genotypes for each estimated variable.

Mann-Whitney U test or Fisher's exact test was used between healthy controls and HCC patients.

Combination 1: Individuals with Ile/Ile of GSTP1 and *A/*A of GSTA.

Combination 2: Individuals with either genotype Ile/Val or Val/Val of GSTP1 or genotype *A/*B or *B/*B of GSTA1.

Combination 3: Individuals with genotype Ile/Val or Val/Val of GSTP1 and genotype *A/*B or *B/*B of GSTA1.

Furthermore, we calculated the AOR and their 95% CIs based on the classification of different age and gender distributions to evaluate the gender- and age-related genetic polymorphism effect on the susceptibility to HCC. Since the mean (±SE) and median age of our 563 recruited subjects was 57.36 ± 0.62 and 57.0 years, respectively, we classified the individuals aged ≤ 57 years as one subgroup and those aged > 57 years as another subgroup. In the younger group, aged ≤ 57 years, individuals with AG or GG alleles of GSTP1 had a 2.18-fold risk (95% CI: 1.09-4.36, p = 0.02) and 5.64-fold risk (95% CI: 1.02-31.18, p = 0.04), respectively, of developing HCC compared to individuals with AA alleles, after adjustment for other confounders, but no association was found in the older group, aged > 57 years (Table 2). No association between the GSTP1 gene polymorphism and HCC susceptibility was found when we further estimated the relationship based on gender grouping. In addition, there was no significant relationship between the GSTA1 gene polymorphism and HCC susceptibility when we estimated it based on different age (Table 2) and gender (Table 3) distributions.

**Table 2:** Adjusted odds ratio (AOR) and 95% confidence intervals (CIs) of hepatocellular carcinoma associated with genotypic frequencies in different age groups.

Variable	Controls n (%)	Patients n (%)	OR (95% CI) p value	AOR (95% CI) p value
	**Age > 57 years old**			

**GSTP1**	**n = 149**	**n = 129**		

II	93 (62.4%)	85 (65.9%)	1.00	1.00

IV	52 (34.9%)	40 (31.0%)	0.84 (0.5-1.3) p = 0.50	0.88 (0.5-1.4) p = 0.63

VV	4 (2.7%)	4 (3.1%)	1.09 (0.2-4.5) p = 0.90	1.06 (0.2-4.6) p = 0.93

**GSTA1**	**n = 149**	**n = 129**		

AA	122 (81.9%)	106 (82.2%)	1.00	1.00

AB	24 (16.1%)	22 (17.1%)	1.05 (0.5-1.9) p = 0.86	1.05 (0.5-2.0) p = 0.87

BB	3 (2.0%)	1 (0.8%)	0.38 (0.03-3.7) p = 0.40	0.47 (0.04-4.68) p = 0.52

	**Age ≤ 57 years old**			

**GSTP1**	**n = 237**	**n = 48**		

II	179 (75.5%)	27 (56.3%)	1.00	1.00

IV	55 (23.2%)	18 (37.5%)	2.17 (1.1-4.2) p = 0.02	2.18 (1.1-4.3) p = 0.02

VV	3 (1.3%)	3 (6.2%)	6.63 (1.2-34.5) p = 0.02	5.64 (1.1-31.8) p = 0.04

**GSTA1**	**n = 237**	**n = 48**		

AA	182 (76.8%)	40 (83.3%)	1.00	1.00

AB	54 (22.8%)	8 (16.7%)	0.67 (0.2-1.5) p = 0.34	0.62 (0.2-1.4) p = 0.28

BB	1 (0.4%)	0 (0%)	- p = 0.99	- p = 0.99

The odds ratios (ORs) with their 95% CIs were estimated by logistic regression models.

The adjusted odds ratios (AORs) with their 95% CIs were estimated by multiple logistic regression models, after controlling for gender and genotypes for each estimated variable.

**Table 3:** Adjusted odds ratio (AOR) and 95% confidence intervals (CIs) of hepatocellular carcinoma associated with genotypic frequencies in different gender groups.

Variable	Controls n (%)	Patients n (%)	OR (95% CI) p value	AOR (95% CI) p value
	**Male**			

**GSTP1**	**n = 257**	**n = 128**		

II	179 (69.7%)	76 (59.4%)	1.00	1.00

IV	72 (28.0%)	47 (36.7%)	1.53 (0.9-2.4) p = 0.06	1.41 (0.8-2.2) p = 0.14

VV	6 (2.3%)	5 (3.9%)	1.96 (0.5-6.6) p = 0.27	1.95 (0.5-6.7) p = 0.29

**GSTA1**	**n = 257**	**n = 128**		

AA	200 (77.8%)	107 (83.6%)	1.00	1.00

AB	53 (20.6%)	20 (15.6%)	0.70 (0.4-1.2) p = 0.22	0.76 (0.4-1.3) p = 0.35

BB	4 (1.6%)	1 (0.8%)	0.46 (0.05-4.2) p = 0.49	0.45 (0.04-4.2) p = 0.49

	**Female**			

**GSTP1**	**n = 129**	**n = 49**		

II	93 (72.1%)	36 (73.5%)	1.00	1.00

IV	35 (27.1%)	11 (22.4%)	0.81 (0.3-1.7) p = 0.60	0.57 (0.2-1.5) p = 0.28

VV	1 (0.8%)	2 (4.1%)	5.16 (0.4-58.7) p = 0.18	3.67 (0.1-107.8) p = 0.45

**GSTA1**	**n = 129**	**n = 49**		

AA	104 (80.6%)	39 (79.6%)	1.00	1.00

AB	25 (19.4%)	10 (20.4%)	1.06 (0.4-2.4) p = 0.87	1.70 (0.5-5.3) p = 0.36

BB	0 (0%)	0 (0%)	-	-

The odds ratios (ORs) with their 95% CIs were estimated by logistic regression models.

The adjusted odds ratios (AORs) with their 95% CIs were estimated by multiple logistic regression models, after controlling for age and genotypes for each estimated variable.

To exclude potential selection biases in estimating the genetic effect on HCC risk, the AOR and 95% CIs of genotypic frequencies and environmental risk factors, such as alcohol, tobacco consumption, detection of hepatitis B surface antigen (HBsAg) and antibody for HCV (anti-HCV), and history of liver cirrhosis of HCC patients were estimated. There was a non-significant difference in distribution between those risk factors and genetic distributions in our recruited HCC patients. The AORs with their 95% CIs of GSTP1 and GSTA1 gene polymorphisms on the clinico-pathological characteristics, such as clinical stage, tumor size, lymph node metastasis, distant metastasis, and Child-Pugh grade in HCC patients were also estimated. We found no association between those estimated clinico-pathological characteristics and gene polymorphisms of GSTP1 and GSTA1. Furthermore, we estimated the relationship between genotypic frequencies and expression levels of clinical pathological markers, such as AFP, AST, and ALT, as well as the ratio of AST to ALT in HCC patients. Similarly, non-significant associations between genetic polymorphisms of GSTP1 and GSTA1 and clinico-pathological markers were found (data not shown).

## Discussion

To the best of our knowledge, this is the first study to provide novel information of GSTP1 and GSTA1 genetic polymorphism effects on HCC risk in Taiwanese.

In this study, based on a 95% CI and p value of 0.05, the ratio of cases to healthy controls was 1:2, our sample size had at least 90% power to detect a 2.0-fold risk in the gene polymorphism of GSTP1 and GSTA1. We found that the allelic frequencies of GSTP1 and GSTA1 were not significantly associated with HCC. McGlynn et al. [34] recruited 231 patients with HCC and 256 healthy controls to examine the association between gene polymorphisms of GSTP1 and GSTA1 and HCC among Chinese who were considered to have been exposed to high levels of aflatoxin B1, a hepatotoxic mycotoxin induced by fungi of the Aspergillus species. They found no association between these two gene polymorphisms and HCC risk, and suggested a non-genetic basis for aflatoxin B1-related susceptibility to HCC risk. Also, Munaka et al. [35] found a non-association between the GSTP1 polymorphism and HCC risk among Japanese. Ladero et al. [36] collected 184 white Spanish patients diagnosed with HCC and 248 healthy controls to estimate the role of the GSTP1 and GSTA1 gene polymorphism on HCC risk; a non-relation between the genetic polymorphism and HCC was found. Although these three studies estimated the relationship between GSTP1 and GSTA1 gene polymorphisms and HCC risk based on different populations with allelic frequencies and environmental exposure different from our recruited population, their results were in agreement with ours. There are other possible explanations for why an individual is not susceptible to HCC, aside from the single nucleotide polymorphisms of GSTP1 and GSTA1, such as linked genes.

However, in the younger group, age ≤ 57 years old, individuals with AG or GG alleles of GSTP1 had a 2.18-fold risk (95% CI: 1.09-4.36, p = 0.02) and 5.64-fold risk (95% CI: 1.02-31.18, p = 0.04) of developing HCC compared to individuals with AA alleles, after adjustment for other confounders, but no association was found in the older group, aged > 57 years. Increased GSTP gene expression was found during early hepatocarcinogenesis by acetylation of histones H3 and H4 and interaction with the specific transcription factor in the promoter regions of the GSTP gene [12]. Moreover, a marked expression of GSTP was found in HCC specimens of HCC patients [13]. It was supposed that normal or increased GSTP1 protein levels or activities contributed to defend normal hepatocytes against a variety of potentially promutagenic stresses [4-6,14,20], assist detoxification, and inhibit mutagenesis [4-6,14,20]. In the present study, the age-related effect of GSTP1 AG and GG alleles on HCC susceptibility in the younger group, aged ≤ 57 years, but not in the older group, aged > 57 years, may have resulted from: 1) The younger group, aged ≤ 57 years, was more likely exposed to HCC-related risk factors [26,29-31]. The G allele of GSTP1 induced the replacement of isoleucine (Ile) with valine (Val) and substantially reduced GSTP1 enzyme activity [24,25], increased the risk of DNA mutation [14,15], and resulted in poor elimination of hydrophilic metabolites [4-6], consequently increasing the susceptibility to HCC when individuals are exposed to carcinogens [9]; 2) Aging was an important risk factor for HCC development [38-40]. The aging effect may have contributed to the non-significant effect of the GSTP1 genetic variant on HCC risk in our older group, because aging-related GSTP1 hyper-methylation was found in normal human tissues [38,39]. This induced the lack of GSTP1 expression [9,16] and may have contributed to carcinogenic development [38,39] and abated the genetic polymorphism effect in the older group. Moreover, different mechanisms contributing to carcinogenesis development were demonstrated in different age groups among other diseases [40,41]. Tremblay et al. [41] recruited 42 young patients (age: 21-40 years) with oral squamous cell carcinomas (OSCCs) and 62 older (age: 60-95 years) OSCCs patients to detect GSTP1 expression in tumor tissues and found a significantly decreased expression of GSTP1 in the tumors of young patients compared to their nondysplastic mucosa, but an increased expression of GSTP1 in tumors of older patients compared with their nondysplastic mucosa [41]. Although those hypotheses need further demonstration in HCC development, we provided a novel finding, that the GSTP1 gene polymorphism increased age-related susceptibility to HCC, particularly for subjects aged ≤ 57 years.

Increased serum levels of AST and ALT are highly correlated with increased expression of GSTA1 [42-44] and GSTP1 [45]. In this study, we estimated the roles of these two gene polymorphisms on the clinical status, such as clinical stage, tumor size, lymph node metastasis, distant metastasis, Child-Pugh grade, and the serum expression levels of liver-related clinical pathological markers, such as alpha-fetoprotein, AST, and ALT, as well as the ratio of AST to ALT in HCC patients. A lack of association between the gene polymorphism and those estimated factors was found in both GSTP1 and GSTA1 genes. We propose that the age-related GSTP1 gene polymorphism contributes to the susceptibility to HCC, and not through the alteration of the expression of those clinical pathological markers.

One of the limitations of our study is that we did not obtain information on risk factors, such as alcohol, tobacco consumption, and other hepatocarcinogenesis-related factors [30] in our recruited healthy control group, because it was difficult to request healthy individuals to provide that information or to collect that data during their health examination. This limitation may impede the adjustment of the confounding variables. However, in order to exclude a potential selection bias in estimating the genetic effect on HCC risk, the AOR and 95% CIs of genotypic frequencies and environmental risk factors, such as alcohol, tobacco consumption, detection of hepatitis B surface antigen (HBsAg) and antibody for HCV (anti-HCV), and the history of liver cirrhosis of HCC patients, were estimated, and there was a non-significant difference in distribution between those risk factors and the genetic distributions in our recruited HCC patients. Moreover, other demographic characteristics such as age, gender, race, and, residential area were adjusted for estimation of gene polymorphism effect on HCC between the healthy controls and HCC patients. We consider that this limitation is unlikely to bound the estimation of genetic effect on HCC risk in this study.

## Conclusion

AG and GG alleles of GSTP1 gene polymorphisms may be considered as factors increasing the susceptibility to the risk of HCC in Taiwanese subjects ages ≤ 57 years.

## Competing interests

The authors declare that they have no competing interests.

## Authors' contributions

YLC: planned the analysis, collected samples, interpreted the data, and drafted the manuscript. HST: analyzed the data and participated in the interpretation of the data. WHK: assisted in the study design and participated in the interpretation of the data. SFY: collected samples, and designed and participated in the analysis of polymerase chain reaction-restriction fragment length polymorphism for measuring the gene polymorphism. DRC: conceived the overall study, secured funding, and revised the manuscript. HTT: conceived the overall study, secured funding, and offered critical revisions to the manuscript. All authors have read and approved the content of the manuscript.

## List of abbreviations

CIs: confidence intervals; GST: glutathione S-transferases; HCC: hepatocellular carcinoma; OR: odds ratios; PCR-RFLP: Polymerase chain reaction-restriction fragment length polymorphism; SNP: single nucleotide polymorphism.

## Pre-publication history

The pre-publication history for this paper can be accessed here:

http://www.biomedcentral.com/1471-2350/11/46/prepub
